# Emerging applications of tumour-educated platelets in the detection and prognostication of ovarian cancer

**DOI:** 10.1093/procel/pwad015

**Published:** 2023-03-27

**Authors:** Jiewei Zhu, Vasily Giannakeas, Steven A Narod, Mohammad R Akbari

**Affiliations:** Women’s College Research Institute, Women’s College Hospital, University of Toronto, Toronto, Ontario M5S 1B2, Canada; Department of Medicine, University of Toronto, Toronto, Ontario M5S 1A, Canada; Women’s College Research Institute, Women’s College Hospital, University of Toronto, Toronto, Ontario M5S 1B2, Canada; Women’s College Research Institute, Women’s College Hospital, University of Toronto, Toronto, Ontario M5S 1B2, Canada; Institute of Medical Science, Faculty of Medicine, University of Toronto, Toronto, Ontario M5S 1A, Canada; Dalla Lana School of Public Health, University of Toronto, Toronto, Ontario M5S 1A, Canada; Women’s College Research Institute, Women’s College Hospital, University of Toronto, Toronto, Ontario M5S 1B2, Canada; Institute of Medical Science, Faculty of Medicine, University of Toronto, Toronto, Ontario M5S 1A, Canada; Dalla Lana School of Public Health, University of Toronto, Toronto, Ontario M5S 1A, Canada

Platelets are nuclear cell fragments produced by megakaryocytes. Inactive platelets circulate in the blood until activation by external stimuli to execute important biological functions, including hemostasis, inflammation, tissue remodelling, and blood vessel formation. Platelets can reflect cancer cells’ pathogenesis through a process known as tumour-educated platelets (TEPs). Education by tumour cells involves three main forms: sequestration of tumour-specific biomolecules (i.e., circulating free proteins, nucleic acids, vesicles, and particles), modulating the splicing of their pre-mRNAs in response to tumour cell signals, which induce tumour-specific splicing events, and megakaryocyte modification to produce TEPs (Sol and Wurdinger, 2017; Dovizio et al., 2020). Several mechanisms have been proposed for the role of TEPs in tumour cell invasion and metastasis, including upregulating cell proliferation and invasion signalling pathways, promoting epithelial-to-mesenchymal transition (EMT) and coating circulating tumour cells (CTC) to allow immune escape (Fig. 1). TEPs are increasingly recognized for their potential use as a readily available liquid biopsy source for earlier cancer detection and prognostication (Zhu et al., 2022; Veld et al., 2022). Two recently published papers in *Protein* & *Cell* emphasized the clinical application of TEPs’ gene expression profiling as a diagnostic and prognostic biomarker in ovarian cancer (Gao et al., 2022; Liu et al., 2022).

**Figure 1.**
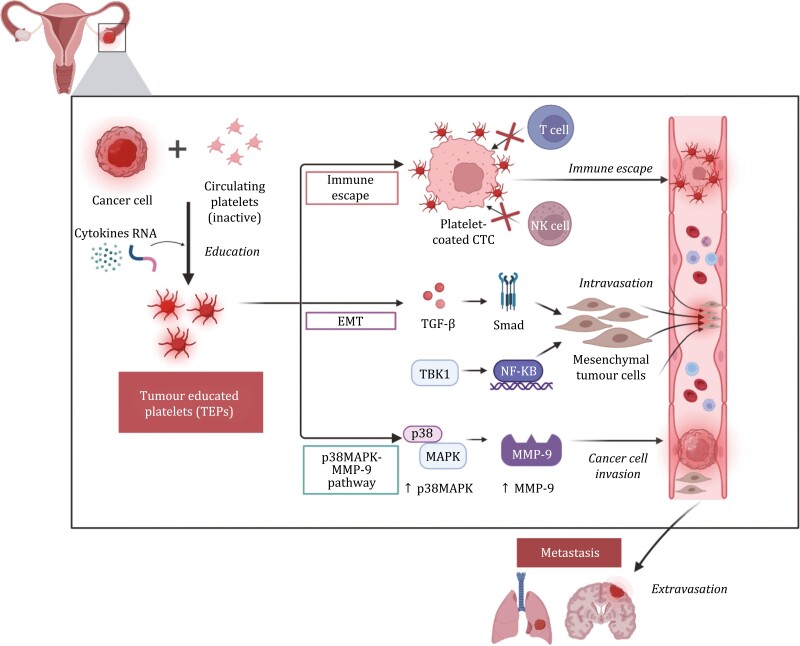
**Three main proposed mechanisms of action for tumour-educated platelets (TEPs).** Cancer cells induce platelet activation through education to generate TEPs. TEPs coat CTCs which promote immune escape, allow tumour cells to intravasate through undergoing EMT and promote tumour cell invasion and metastasis through p38MAPK-MMP-9 pathway regulation. CTC, circulating tumour cell; EMT, epithelial-to-mesenchymal transition; MMP-9, matrix metalloproteinase 9; TBK1, TANK Binding Kinase 1; NF-κB, nuclear factor kappa-light-chain-enhancer of activated B cells.

## New evidence for TEPs in the early diagnosis of ovarian cancer

Previous studies have reported that mRNA sequencing of TEPs can discriminate ovarian cancer patients from healthy controls with 80% accuracy based on small size samples (Piek et al., 2019; Antunes-Ferreira et al., 2021). Building on this evidence, Gao et al. developed a classifier of platelet RNA profiles based on a large sample size (*n* = 520) and evaluated its ability to provide an early and accurate diagnosis of ovarian cancer in samples consisting of two Chinese and one European validation cohorts. Platelet mRNA-seq data were filtered by a feature selection method followed by a minimum redundancy maximum relevance ranking algorithm to generate a model consisting of 102 platelet RNAs, called TEP-derived gene panel of ovarian cancer (TEPOC). Using receiver operating characteristic (ROC) analysis, Gao et al. demonstrated that TEPOC allows accurate diagnosis of ovarian cancer in the combined sample, with an area under the curve (AUC) of 0.918. The study authors then compared the diagnostic performance of TEPOC model to carbohydrate antigen 125 (CA-125). Despite being the best characterized biomarker for ovarian cancer currently, CA-125 is not recommended for routine screening due to its low sensitivity and specificity for early-stage ovarian cancer detection. Subgroup analysis demonstrated that TEPOC alone did not have a significantly higher accuracy for detecting early-stage (stage I–IIA) ovarian cancer compared to CA-125 (AUC 0.858 vs. 0.749; *P* = 0.060). However, combining CA-125 with TEPOC improved the diagnostic performance for early-stage ovarian cancer in the combined samples (AUC = 0.879 for combined vs. AUC = 0.749 for CA-125 alone; *P* = 0.005). Although there was no subgroup analysis for advanced stage ovarian cancer, TEPOC was able to detect high grade serous ovarian cancer with AUC of 0.903.

## Role of TEPs in predicting ovarian cancer prognosis

Thrombocytosis (platelet count > 350 × 10^9^/L) has previously been associated with significantly shorter ovarian cancer survival (Stone et al., 2012). Similarly, the recent paper by Liu et al. (2022) analyzing pre-treatment blood samples of 2,404 ovarian cancer patients also found that thrombocytosis (>350 × 10^9^/L) was significantly associated with shorter overall survival (median survival of 70 months for platelet count > 350 × 10^9^/L group vs. 120 months for platelet count ≤ 350 × 10^9^/L group; log-rank *P* < 0.0001). Further, this study suggested that TEPs’ RNA analysis may allow for personalized ovarian cancer survival prediction. Specifically, Liu et al. performed RNA sequencing of TEPs from 303 ovarian cancer pre-treatment samples to develop a DeepCox model based on a deep learning algorithm for ovarian cancer survival prediction with a platelet signature of 100 genes. The DeepCox model generated a continuous risk score to reflect favourable (low risk) or dismal (high risk) outcomes which showed high concordance with both progression-free survival (*C*-index = 0.891; *P* = 0) and overall survival [*C*-index = 0.713; *P* = 7.73 × 10^−12^ (Chen et al., 2022)]. The DeepCox scores were validated in two cohorts with a median follow-up of 35 months. Time-dependent ROC analysis demonstrated AUC for 5-year overall survival of 0.869 and 0.843 in each of the validation cohorts, respectively. Importantly, higher risk scores were significantly associated with worse progression-free survival [adjusted Hazards Ratio (aHR) = 6.83; 95% CI: 4.97–9.39; *P* < 0.001] and overall survival (aHR = 4.13; 95% CI: 2.79–6.11; *P* < 0.001) after adjusting for other factors. To date, the DeepCox model is the only prognosis model for ovarian cancer generated by deep learning algorithm based on platelet RNAs.

## Applications of TEPs in ovarian cancer

Although there is emerging research supporting TEPs as an important bio-source for liquid biopsies (Table S1), robust evidence for its routine application in earlier ovarian cancer diagnosis is lacking. Ovarian cancer typically evades early detection due to its insidious symptoms and presents with widespread advanced disease at diagnosis with poor prognosis. Over 75% of disease is diagnosed in the advanced stage with a 5-year survival rate of 29% compared with 92% for localized disease (Siegel et al., 2021). Although detecting early-stage may improve the prognosis of ovarian cancer, there is currently a large unmet need for diagnostic biomarkers as no screening tools have been proven effective to date (Siegel et al., 2021). Studies have examined the diagnostic performance of other liquid biopsy components, including CTCs (79.4% sensitivity and 92.2% specificity) (Wang et al., 2022), exosomal miRNAs (model AUC = 0.8337) (Chen et al., 2022), and ctDNA (sensitivity = 88%, specificity 80%) (Passiglia et al., 2018; Sabatier et al., 2022). Major advantages of TEPs include the extensive interactions between platelets and tumour cells, high availability in the peripheral blood, lack of interference from genomic DNA given their anuclear structure, and ease of isolation. Further, the long latency of ovarian cancer enables extensive tumour–platelet interactions that increase the chances of generating TEPs and intensify the molecular abnormalities of TEPs, which provide opportunities to leverage TEPs in ovarian cancer. A recent study by Veld et al. demonstrated that TEP’s RNA analysis enables early cancer detection and identification of the tumour site of origin for 18 different cancers (Veld et al., 2022). By using a panel of 492 platelet RNA biomarkers, the ThromboSeq prediction algorithm had an overall sensitivity of 64% and specificity of 99%. For ovarian cancer, the overall sensitivity was 59%, with higher sensitivity for more advanced stages (stage I = 48%, stage II = 50%, stage III = 58% and stage IV = 69%) (Veld et al., 2022). Another study reported that for early-stage ovarian cancer (stage I–II), multivariate prediction modelling based on platelet protein expression profiles correctly predicted seven out of eight cases of ovarian cancer with a sensitivity of 83% and specificity of 76% (AUC = 0.831, *P* < 0.0001) (Lomnytska et al., 2018). The diagnostic accuracy of TEPOC in the study by Gao et al. was comparable or higher and supports the utility of TEPs-derived RNA analysis for earlier detection of ovarian cancer. Importantly, this study also suggests that CA-125 in combination with TEPs as biomarker may improve the currently limited sensitivity and specificity of CA-125 as a screening modality for ovarian cancer. Strengths of this study include validation of TEPOC among different ethnicities and histological subtypes. However, although the TEPOC model demonstrated good predictive accuracy in the analytic cohorts, more than half of enrolled patients had advanced ovarian cancer. Future prospective, population-based studies with a larger sample of early-stage ovarian cancer cases are required. Another limitation of TEP application is the widely varied assay methods used across studies, which requires standardization prior to introducing TEPs as a routine non-invasive diagnostic modality in the clinical setting. Lastly, the study by Gao et al. and most studies to date use bulk analysis approaches where platelets are indiscriminately isolated from whole blood before lysis and sequencing. With more advanced technologies, future studies with single-cell RNA sequencing of platelets can elucidate whether platelet education by tumour cells is homogenous and allow more accurate detection of heterogeneous tumours (Psaila et al., 2020).

Prognosis prediction in ovarian cancer is very challenging due to the remarkable molecular and histological heterogeneity. Effective prognostic biomarkers in clinical practice are lacking and there is an urgent need to identify prognostic biomarkers independent of molecular and pathological types. Both increased platelet count and specific platelet RNA signature may be associated with worse ovarian cancer survival. Giannakeas et al. (2022) reported that cancer-specific mortality was much higher for stage I/II ovarian cancer patients with a high (≥75th percentile) platelet count compared to those with medium (>25th and <75th percentile) platelet counts (aHR 3.10; 95%CI 1.74–5.52), with a 5-year survival for low/medium platelet count patients (<75th percentile) of 91% compared to 71% for those with high platelet count. Similarly, the study by Liu et al. also supports that thrombocytosis may be a prognostic indicator associated with worse overall survival. Further, this study suggests that pre-treatment platelet RNA may allow for mortality risk stratification in ovarian cancer patients. Although the survival prediction results were promising in the two validation cohorts, more data is required for validation in larger and more diverse populations.

Both studies provide evidence for TEPs as a versatile liquid biopsy component that addresses the current clinical challenges in providing accurate diagnosis and prognostication in ovarian cancer. Compared to previous TEP-based models, a key advantage of TEPOC and DeepCox is the small scale of models that increases its clinical utility, including around 100 genes compared to over 1000 in a previous study by Best et al. (Antunes-Ferreira et al., 2021) or approximately 500 in ThromboSeq (Veld et al., 2022). Another strength of TEPOC is its derivation from an international, sample size-enhanced study including the largest sample of ovarian cancer patients to date and validation across different ethnicities, heterogeneous histological subtypes, and early-stage ovarian cancer that demonstrated good robustness and compatibility for preoperative diagnosis of ovarian cancer. Meanwhile, DeepCox is the first TEPs-enabled cancer prognosis prediction model to date and suggests the possible utility of TEPs RNAs in providing more personalized prognostication in ovarian cancer. As such, both studies provide data supporting the clinical potential and application significance of TEPs.

## Supplementary Material

pwad015_suppl_Supplementary_Material

## References

[CIT0001] Antunes-Ferreira M , Koppers-Lalic D, Würdinger T. Circulating platelets as liquid biopsy sources for cancer detection. Mol Oncol 2021;15:1727–1743. 33219615 10.1002/1878-0261.12859 PMC8169446

[CIT0002] Veld SGJG , Arkani M, Post E et al. Detection and localization of early- and late-stage cancers using platelet RNA. Cancer Cell 2022;40:999–1009.e6. 36055228 10.1016/j.ccell.2022.08.006

[CIT0003] Chen L , Wang K, Li L et al. Plasma exosomal miR-1260a, miR-7977 and miR-192-5p as diagnostic biomarkers in epithelial ovarian cancer. Future Oncol 2022;18:2919–2931. 35893704 10.2217/fon-2022-0321

[CIT0004] Dovizio M , Ballerini P, Fullone R et al. Multifaceted functions of platelets in cancer: from tumorigenesis to liquid biopsy tool and drug delivery system. Int J Mol Sci 2020;21:9585. 33339204 10.3390/ijms21249585 PMC7765591

[CIT0005] Gao Y , Liu CJ, Li HY et al. Platelet RNA enables accurate detection of ovarian cancer: an intercontinental, biomarker identification study. Protein Cell 2022. 10.1093/procel/pwac056 PMC10246718 36905391

[CIT0006] Giannakeas V. Trends in platelet count among cancer patients. Exp Hematol Oncol 2022;11:16. 35331331 10.1186/s40164-022-00272-3 PMC8944120

[CIT0007] Liu CJ , Li HY, Gao Y et al. Platelet RNA signature independently predicts ovarian cancer prognosis by deep learning neural network model. Protein Cell 2022. 10.1093/procel/pwac053 PMC10392027 37526343

[CIT0008] Lomnytska M , Pinto R, Becker S et al. Platelet protein biomarker panel for ovarian cancer diagnosis. Biomark Res 2018;6:2. 29344361 10.1186/s40364-018-0118-y PMC5767003

[CIT0009] Passiglia F , Rizzo S, Di Maio M et al. The diagnostic accuracy of circulating tumor DNA for the detection of EGFR-T790M mutation in NSCLC: a systematic review and meta-analysis [published correction appears in Sci Rep. 2018 Nov 19;8(1):17270]. Sci Rep 2018;8:13379. 30190486 10.1038/s41598-018-30780-4 PMC6127187

[CIT0010] Piek J , In ’t Veld S, Best M et al. EP457 assessment of ovarian tumors with tumor-educated platelets (TEPs). Int J Gynecol Cancer 2019;29:A291.

[CIT0011] Psaila B , Wang G, Rodriguez-Meira A et al.; NIH Intramural Sequencing Center. Single-cell analyses reveal megakaryocyte-biased hematopoiesis in myelofibrosis and identify mutant clone-specific targets. Mol Cell 2020;78:477–492.e8. 32386542 10.1016/j.molcel.2020.04.008 PMC7217381

[CIT0012] Sabatier R , Garnier S, Guille A et al. Whole-genome/exome analysis of circulating tumor DNA and comparison to tumor genomics from patients with heavily pre-treated ovarian cancer: subset analysis of the PERMED-01 trial. Front Oncol 2022;12:946257. 35965534 10.3389/fonc.2022.946257 PMC9373051

[CIT0013] Siegel RL , Miller KD, Fuchs HE et al. Cancer Statistics, 2021. CA Cancer J Clin 2021;71:7–33. 33433946 10.3322/caac.21654

[CIT0014] Sol N , Wurdinger T. Platelet RNA signatures for the detection of cancer. Cancer Metastasis Rev 2017;36:263–272. 28681241 10.1007/s10555-017-9674-0 PMC5557864

[CIT0015] Stone R , Nick A, McNeish I et al. Paraneoplastic thrombocytosis in ovarian cancer. N Engl J Med 2012;366:610–618. 22335738 10.1056/NEJMoa1110352 PMC3296780

[CIT0016] Wang T , Gao Y, Wang Xi et al. Establishment of an optimized CTC detection model consisting of EpCAM, MUC1 and WT1 in epithelial ovarian cancer and its correlation with clinical characteristics. Chin J Cancer Res 2022;34:95–108. 35685992 10.21147/j.issn.1000-9604.2022.02.04 PMC9086574

[CIT0017] Zhu JW , Charkhchi P, Akbari MR. Potential clinical utility of liquid biopsies in ovarian cancer. Mol Cancer 2022;21:114. 35545786 10.1186/s12943-022-01588-8 PMC9092780

