# Supplementary material for: Emerging applications of tumour-educated platelets in the detection and prognostication of ovarian cancer
Source: Protein Cell. 2023 Mar 27;14(8):556–9. doi: 10.1093/procel/pwad015 (PMC10392028; doi:10.1093/procel/pwad015)
Supplement: pwad015_suppl_Supplementary_Material [file pwad015_suppl_supplementary_material.pdf]

Supplementary Table 1. Previous studies examining TEPs in ovarian cancer

Study	TEPs biomarker	Year	Participants	Clinical significance of biomarker	Clinical application	Key findings
Lomnytska et al.[17]	35 platelet proteins	2018	57 cases of benign adnexal lesions, 49 cases of stage III/IV ovarian cancer, 8 cases of stage I/II ovarian cancer	Functional relevance to ovarian cancer, normal platelet biology and platelet-associated pathological conditions	Diagnosis	Platelet protein expression can differentiate between advanced ovarian cancer vs benign adnexal lesions with 96% sensitivity and 88% specificity. Model correctly predicted 7/8 cases of early-stage ovarian cancer with sensitivity of 83% and specificity of 76% (AUC) = 0.831, $p < 0.0001$)
Mysona et al.[19]	1129 TEP proteins	2019	71 HGSOC	Serum concentration of TEP proteins at remission	Prognosis	Brain derived neurotrophic factor and platelet-derived growth factor molecules were significant for predicting progression-free survival on both univariate and multivariate analyses
Battaglia et al.[20]	Circulating PD-L1+ microvesicles (MVs) of platelet origin	2021	63 HGSOC patients	Surface PD-L1 as proxy for the whole tumour PD-L1 status with potential benefit from immunotherapy	Treatment	Enumeration of circulating PD-L1+ MVs may enable assessment of tumour PD-L1 status and stratification of HGSOC patients for immunotherapy interventions

Piek et al.[18]	TEPs RNA	2019	Training cohort = 41 women (20 stage I/II ovarian cancer and 21 healthy controls)<br><br>Evaluation cohort = 43 women (21 ovarian cancer patients, and 20 benign tumours)	-	Diagnosis	TEPs can differentiate early-stage ovarian cancer from benign pathologies with 80% accuracy
Veld et al.[4]	TEP RNA	2022	144 ovarian cancer patients	-	Diagnosis and localization	AUC = 0.89 (95% CI: 0.84–0.93)<br><br>Overall prediction rate = 59% (95% CI: 0.48– 0.68)<br><br>Prediction rate by stage:<br>Stage I = 48% (95%CI: 0.28– 0.68)<br>Stage II = 50% (95%CI: 0.18– 0.81)<br>Stage III = 58% (95%CI: 0.40– 0.74) 6<br>Stage IV = 69% (95%CI: 0.50– 0.83; 32)

*HGSOC = high grade serous ovarian cancer
